# Hyperinflammation in Two Severe Acute Respiratory Syndrome Coronavirus 2-Infected Adolescents Successfully Treated With the Interleukin-1 Inhibitor Anakinra and Glucocorticoids

**DOI:** 10.3389/fped.2020.576912

**Published:** 2020-11-30

**Authors:** Francesca I. Calò Carducci, Maria Antonietta De Ioris, Chiara Agrati, Rita Carsetti, Daniela Perrotta, Patrizia D'Argenio, Fabrizio De Benedetti, Stefania Notari, Paolo Rossi, Andrea Campana

**Affiliations:** ^1^Academic Department of Pediatrics, Bambino Gesù Children's Hospital, IRCCS, Rome, Italy; ^2^Cellular Immunology Laboratory, IRCCS National Institute for Infectious Diseases “L. Spallanzani”, Rome, Italy; ^3^Department of Pediatric Hematology and Oncology, Bambino Gesù Children's Hospital, IRCCS, Rome, Italy; ^4^Pediatric Intensive Care Unit, Bambino Gesù Children's Hospital, IRCCS, Rome, Italy; ^5^Division of Rheumatology, Bambino Gesù Children's Hospital, IRCCS, Rome, Italy

**Keywords:** pediatric, coronavirus disease 2019, MIS-C, PIMS-TS, cytokine storm, anti-inflammatory treatment, SARS-CoV-2, COVID-19

## Abstract

**Background:** In severe acute respiratory syndrome-related coronavirus (SARS-CoV-2) critically ill adults, hyperinflammation plays a key role in disease progression. The clinical manifestations of SARS-CoV-2 infection among children are much less severe compared with adult patients and usually associated with a good prognosis. However, hyperinflammation in SARS-CoV-2-infected pediatric patients has been described as pediatric inflammatory multisystem syndrome temporally associated with SARS-CoV-2 or as Kawasaki-like disease but is still little known, and optimal management has to be defined. The World Health Organization (WHO) on the 15th of May 2020 has developed a preliminary case definition for multisystem inflammatory disorder in children and adolescents with coronavirus disease 2019 (COVID-19) and stated for an urgent need to collect data on this condition. Here, we report two adolescent patients affected by COVID-19 presenting with multisystem inflammatory disorder, 3–4 weeks after the first symptoms of SARS-CoV-2 infection, treated with the interleukin-1 receptor antagonist anakinra and glucocorticoids with good clinical response.

**Cases:** We report two patients chronically ill appearing, with high fever, severe gastrointestinal involvement, and increased biomarkers of inflammation onset 3–4 weeks after paucisymptomatic SARS-CoV-2 infection. They had no lung involvement, but abdominal ultrasound and CT scan showed thickening of the bowel wall. SARS-CoV-2 PCR was positive on ileum biopsy in both patients, whereas it was negative on other common sampled sites. They have been admitted to the pediatric intensive care unit and have been treated with a combination of anakinra 6–8 mg/kg/day i.v. and a standard dose of methylprednisolone 2 mg/kg/day in addition to lopinavir/ritonavir 400 mg q12h and low molecular weight heparin 100 UI/kg q12h with good clinical response.

## Background

The outbreak of a novel coronavirus infection—severe acute respiratory syndrome coronavirus 2 (SARS-CoV-2)—has rapidly spread worldwide since its onset in December 2019. The World Health Organization (WHO) on the 23rd of October 2020 registered 41,570,883 confirmed cases and 1,134,940 coronavirus disease 2019 (COVID-19)-related deaths worldwide (1). In critically ill adult patients, hyperinflammation is considered to play a major causative role in the pathological process leading to acute respiratory distress syndrome (ARDS) and multiple-organ failure (2, 3). The clinical manifestations of SARS-CoV-2 infection among children are much less severe compared with adult patients and usually associated with a good prognosis (4, 5). Although COVID-19 is most well-known for causing substantial respiratory pathology, it can also result in several extrapulmonary manifestations such as thrombotic complications and myocardial, kidney, gastrointestinal, hepatocellular, neurologic, cutaneous, and ocular injury. Direct viral tissue damage is a plausible mechanism of injury. In addition, endothelial damage and dysregulation of immune responses might contribute to these extrapulmonary manifestations (6, 7). Hyperinflammation in SARS-CoV-2-infected pediatric patients has been described as pediatric inflammatory multisystem syndrome temporally associated with SARS-CoV-2 (8) or as Kawasaki-like disease (9) or as multisystem inflammatory syndrome in children (MIS-C) (10) but is still little known, and optimal management has to be defined. The WHO on the 15th of May 2020 has developed a preliminary case definition for multisystem inflammatory disorder in children and adolescents with COVID-19 and stated that there is an urgent need to collect data on this condition (11).

Here, we report two adolescent patients affected by COVID-19 presenting with hyperinflammation, 3–4 weeks after the first symptoms of SARS-CoV-2 infection, treated with the interleukin (IL)-1 receptor antagonist anakinra and glucocorticoids with good clinical response.

## Case Presentation

Patient 1 is a 14-year-old boy (weight 50 kg) affected by celiac disease, whose mother tested positive for SARS-CoV-2. The boy presented with a 12-day history of mild dry cough and low-grade fever; SARS-CoV-2 was detected by means of real-time polymerase chain reaction (RT-PCR) on nasopharyngeal swab, and the patient was referred to our hospital because of persisting fever. At admission, general conditions were good, oxygen saturation level was 98% on room air, and body temperature was normal. Blood examinations, chest X-ray, and electrocardiogram (EKG) were normal. On day 2, SARS-CoV-2 RT-PCR was negative on nasopharyngeal and conjunctival swabs and on fecal and urine samples and was repeated every 72 h with the same results (Table 1).

**Table 1:** Demographics, time from the first symptoms, and microbiological findings.

	**Patient 1**	**Patient 2**
**Demographics**	14 years old (55 kg, BMI 16.98 kg/m^2^), male Caucasian	13 years old (70 kg, BMI 26.45 kg/m^2^), male Caucasian
**Time from the first symptoms of infection**	21 days	27 days
**Microbiological findings**	**ICU admission**	**ICU admission**
Nasopharyngeal swab SARS-CoV-2 PCR	Negative	Negative
Conjunctival swab SARS-CoV-2 PCR	Negative	Negative
Stool SARS-CoV-2 PCR	Negative	Negative
Urine SARS-CoV-2 PCR	Negative	Negative
Ileum biopsy SARS-CoV-2 PCR	Positive	Positive
SARS-CoV-2 IgA	1:160	1:320
SARS-CoV-2 IgM	1:40	1:40
SARS-CoV-2 IgG	1:80	1:640

On day 4 after admission, he developed fever (39.5°C), abdominal pain, and diarrhea. Lung CT scan and echocardiography were normal. Due to persisting gastrointestinal symptoms, abdominal ultrasound, and CT scan were performed and showed widespread thickening of the distal loops of the small intestine with a small amount of ascitic fluid. Due to the worsening of general conditions, the boy was admitted in our pediatric intensive care unit (PICU). Bone marrow biopsy showed neither hematologic malignancies nor activated macrophages. Colonscopy with retrograde ileoscopy showed no macroscopic lesion. Histological examination of multiple intestinal biopsies showed minimal inflammatory infiltrates. SARS-CoV-2 PCR was positive on ileum biopsy. Serological test showed SARS-CoV-2 IgA 1:160, IgG 1:80, and IgM 1:40. Microbiological workout was negative, including blood, throat, sputum, urine, and stool culture; respiratory and stool viral panel, blood viral panel including human immunodeficiency virus, hepatitis B virus, hepatitis C virus, cytomegalovirus, Epstein–Barr virus, parvovirus B19, herpes simplex virus 1 and 2, human herpes virus 6 and adenovirus, and interferon γ release assay (QuantiFERON®-TB gold).

On day 9, blood chemistry showed a significant increase of inflammatory markers, D-dimers, and troponin T, associated with lymphopenia (Table 2). Treatment with methylprednisolone 2 mg/kg/day and anakinra 100 mg q6h i.v. was started; in addition, the child was given treatment with lopinavir/ritonavir 400 mg q12h and broad spectrum antibiotics plus low molecular weight heparin (LMWH) 100 UI/kg q12h. Twenty-four hours later, fever disappeared and general conditions significantly improved.

**Table 2:** Laboratory findings and cytokine levels.

		**Patient 1**		**Patient 2**	
**Laboratory findings**	**Normal range**	**ICU admission**	**Day 14 of treatment**	**ICU admission**	**Day 12 of treatment**
WBC count per mm^3^	4,000–13,500	5,000	10,560	12,440	9,790
Lymphocyte count per mm^3^	1,040–6,480	500	2,230	990	1,800
Hemoglobin (g/dL)	10.5–15.5	10.7	11.6	10.4	10.4
Platelet per mm^3^	150,000–450,000	116,000	293,000	186,000	371,000
C-reactive protein (mg/dL)	<0.5	19	0.1	28	0.14
Procalcitonin (ng/mL)	<0.5	7.9	0.04	1.2	0.03
Erythrocyte sedimentation rate in mm	0–15	57	2	74	4
Troponin T high sensitive (ng/L)	<14	86.5	5.2	173	57
N-terminal prohormone of brain natriuretic peptide (pg/mL)	<186	812	48.6	190	41
d-dimer (mg/L)	<0.5	5.42	0.37	>20	0.33
Fibrinogen (mg/dL)	212–433	564	237	643	213
Ferritin (μg/mL)	30–400	772	386	419	227
Lactic dehydrogenase (U/L)	120–300	365	189	351	227
Serum albumin (g/dL)	3.8–5.4	3.3	4.6	4.0	3.5
Sodium (mEq/L)	136–145	135	135	137	138
Potassium (mEq/L)	3.1–5.1	4.0	4.7	4.25	4.02
IL-1β (pg/mL)	<0.04	1.281	0.37	2.505	0.36
IL-6 (pg/mL)	0.56–2.74	76.4	0.75	50.32	0.68
IL-8 (pg/mL)	1.39–12.68	40.919	36.31	209.656	22.33
TNF-α (pg/mL)	2.87–9.39	27.334	6.41	20.859	5.62

Patient 2 is a 13-year-old boy (weight 70 kg); the grandfather died due to SARS-CoV-2 pneumonia. Two days later, he presented fever (39.3°C) and dry cough; SARS-CoV-2 PCR was positive on nasopharyngeal swab and he was referred to our center.

At admission, general conditions were goo and oxygen saturation was 98% in room air; blood test, chest X-ray, and EKG were normal. Fever and cough disappeared after 24 h and he was discharged 9 days later. Serological test at discharge showed SARS-CoV-2 IgA 1:320, IgG 1:640, and IgM 1:40. Ten and 13 days after discharge, SARS-CoV-2 nasopharyngeal swabs were negative (Table 1).

Sixteen days after discharge, he presented fever (39.5°C), abdominal pain, vomiting, and diarrhea and was readmitted. SARS-CoV-2 PCR was negative on nasopharyngeal and conjunctival swabs and on fecal and urine samples and was repeated every 72 h with the same results. Chest X-ray and CT scan, EKG, and echocardiography were normal. Microbiological workout was negative. Seventy-two hours later, due to the worsening of general conditions and gastrointestinal symptoms, abdominal ultrasound and CT scan were performed and showed thickening of the bowel wall. Colonscopy with retrograde ileoscopy showed no macroscopic lesions. Histological examination of multiple intestinal biopsies showed minimal non-specific inflammatory infiltrates. SARS-CoV-2 PCR was positive on ileum biopsy. Bone marrow biopsy showed neither hematologic malignancies nor activated macrophages. Blood chemistry showed significant increase of inflammatory markers, D-dimers, and troponin T, associated with lymphopenia and anemia; he was admitted to the PICU and treatment with methylprednisolone 2 mg/kg/day and anakinra 100 mg q6h i.v. was started, in addition to lopinavir/ritonavir 400 mg q12h, broad spectrum antibiotics, and LMWH 100 UI/kg q12h. Twenty-four hours later, fever disappeared and general conditions significantly improved.

Both patients experienced asymptomatic bradycardia with elongation of QT interval managed with magnesium i.v. and discontinuation of all drugs involved in QT tract elongation with complete resolution. Moreover, the increase in troponin T and BNP normalized during the treatment.

The two patients have been discharged from the PICU (day 14 and day 10, respectively) with complete resolution of symptoms; blood abnormalities were significantly improved or normalized (Table 2).

**Figure 1:**
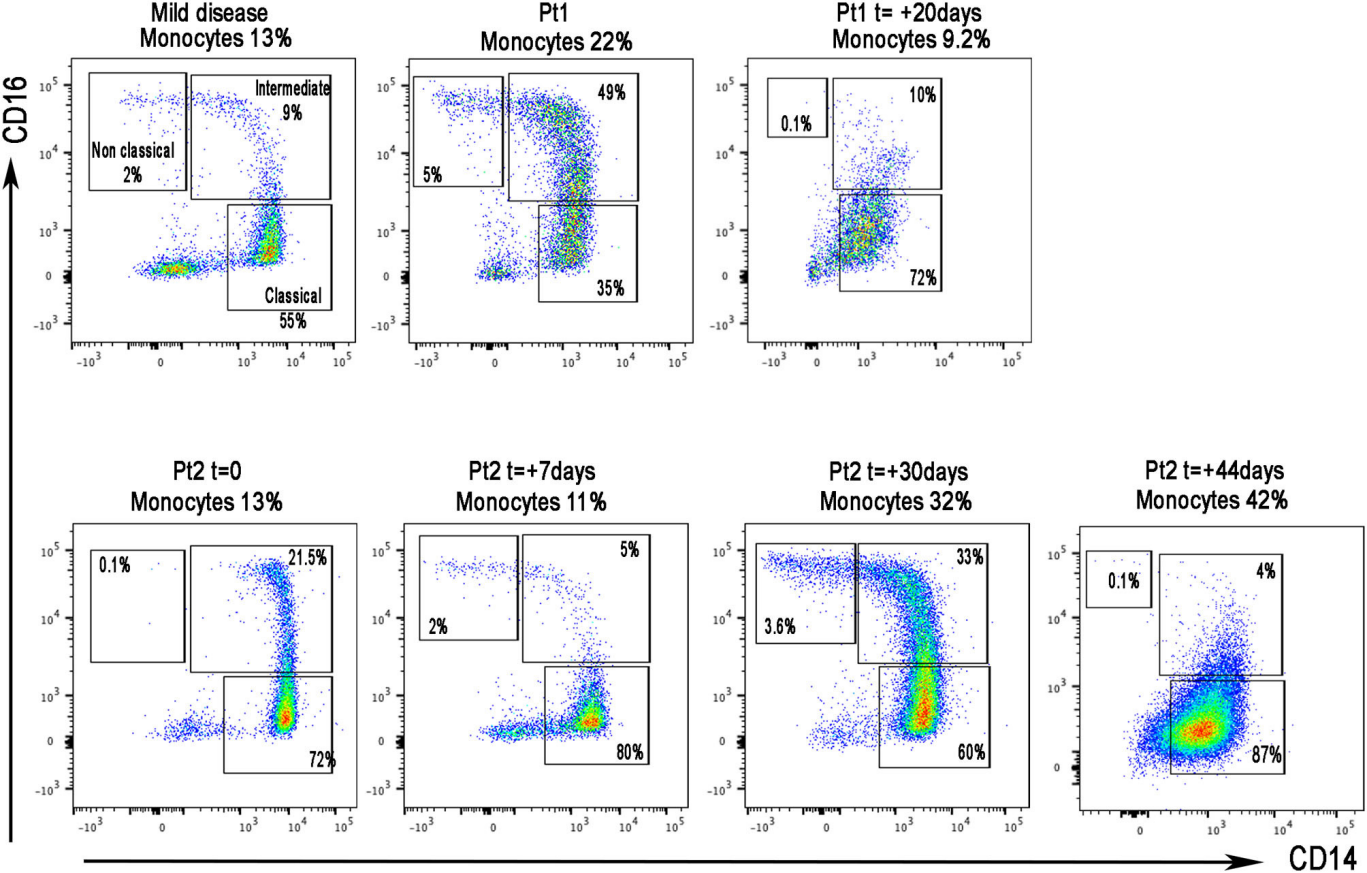
Flow cytometric analysis of monocytes in the peripheral blood in three patients diagnosed as positive for SARS-CoV-2: one with mild disease and two with signs of severe cytokine storm. Monocytes were identified as CD3^−^ large cells gated based on HLA-DR and CD14 expression. Monocytes were further classified as classical (CD14^+^CD16^−^), intermediate (CD14^+^CD16^+^), and non-classical (CD14^−^CD16^+^).

In both patients, flow cytometry analysis of peripheral blood mononuclear cells showed a high monocyte count (patient 1, 22%; patient 2, 32%), due to the increase in intermediate and non-classical monocytes, which are known to produce inflammatory cytokines (Figure 1) (12); IL-1β, IL-6, IL-8, and tumor necrosis factor-alpha (TNF-α) serum levels were elevated (Table 2). After 14 days of treatment, monocyte count (Figure 1) and interleukin levels (Table 2) were significantly decreased.

At present both patients are at home, off-therapy, without symptoms.

## Discussion

Clinical findings in adults showed that hyperinflammation and cytokine storm seem to drive the pathophysiological mechanism leading to multiorgan failure (2, 3). The exaggerated inflammatory response to SARS-CoV-2 promotes the occurrence of ARDS, disseminated intravascular coagulation, and myocardial damages, which are the main players determining fatalities (13). The pediatric presentation of COVID-19 disease was reported to be mild, with exceptional deaths (4, 5). Recently, a new syndrome called pediatric inflammatory multisystem syndrome temporally associated with SARS-CoV-2 (PIMS-TS) has been described (8). S. Riphagen and colleagues reported nine children with PIMS-TS (14). A few days later, Verdoni et al. described an outbreak of 10 patients with a severe Kawasaki-like disease after the SARS-CoV-2 epidemic (9). In both case series, children were administered intravenous immunoglobulin and some received also aspirin and/or glucocorticoids.

E. Whittaker and colleagues (15) reported 58 patients with PIMS-TS: 71% were treated with intravenous immunoglobulin, 64% with corticosteroids, 14% with TNF-α antagonist infliximab, and 5% with the IL-1 receptor antagonist anakinra. Our cases presented with high fever, poor general conditions, and increased biomarkers of inflammation with clinical picture dominated by severe gastrointestinal symptoms. The patients showed lymphopenia, marked increase in CRP, and increase in LDH, ferritin, and D-dimers, features described in PIMS-TS patients as well as in other forms of hyperinflammation such as hemophagocytic lymphohistiocytosis (HLH) secondary to infections. Moreover, both showed myocardial injury, as demonstrated by elevation of troponin T levels and a significant increase in monocytes producing inflammatory cytokines and in serum levels of inflammatory cytokines. As these features have been shown to be associated with both severe and fatal COVID-19 in adults (12, 16, 17), we chose to treat with the combination of anakinra 6–8 mg/kg/day i.v. and a standard dose of methylprednisolone 2 mg/kg/day. Anakinra is an IL-1 receptor antagonist widely used in children with systemic juvenile idiopathic arthritis and other autoinflammatory diseases (18). The safety profile of anakinra has been consistent across indications, age groups, and doses studied. High doses of anakinra have been reported to be efficacious in different forms of hyperinflammation (19), and the dosing regimen chosen is being used in the ongoing trial of COVID-19 (NCT04324021). In the setting of an active viral infection with the goal to interfere as little as possible with viral clearance, we deemed it appropriate to use a standard dose of glucocorticoids instead of high-dose pulses (up to 30 mg/kg/day) that are being used typically in hemophagocytic lymphohistiocytosis and other forms of hyperinflammation. The clinical response to this combination was satisfactory, with complete control of symptoms in 24 h and progressive improvement of chemistry, monocytes, and cytokine levels.

Notably, in the adult population, COVID-19 disease is mainly dominated by fever and pneumonia, with severe cases showing cytokine storm that leads to ARDS (2, 3), whereas in our pediatric patients, there was no lung involvement. Nevertheless, COVID-19 can also result in several extrapulmonary manifestations such as thrombotic complications and myocardial, kidney, gastrointestinal, hepatocellular, neurologic, cutaneous, and ocular injury (6, 7).

Gastrointestinal (GI) symptoms are present in COVID-19 patients in a percentage as high as 50%: nausea, diarrhea, anorexia, abdominal pain, belching, and emesis can occur. Although the pathogenesis is still being investigated, direct viral tissue damage is a plausible mechanism of injury *via* the angiotensin-converting enzyme 2 (ACE2) protein. Another potential mechanism can involve proinflammatory cytokines that can alter the gut–brain axis and gut flora, due to the use of antimicrobials, concomitant infections, and the severe illness itself (20, 21). Once SARS-CoV-2 enters the GI tract by oral contact (22) or by ingestion of lung secretions, it can cause viral cytopathy in areas with high prevalence of ACE2. Hence, the ileum is one of the most common sites involved (23, 24).

Our patients presented with a clinical picture characterized by hyperinflammation and gastrointestinal symptoms; thus, we hypothesized that SARS-CoV-2 could have triggered an inflammatory bowel disease; consequently, we performed colonoscopy with ileoscopy. Macroscopic and histological examination turned out negative, suggesting a direct pathogenic role of the virus, consistent with the detection of SARS-CoV-2 in ileum biopsies. We want to underline that we do not suggest to perform gastrointestinal endoscopy to all COVID-19 patients with gastrointestinal symptoms. The risk–benefit analysis must be performed on each case taking in mind the risk of infection and transmission related to this procedure in active SARS-CoV-2 disease (25).

Notably, hyperinflammation occurred 3–4 weeks after the first symptoms of SARS-CoV-2 infection, thus suggesting, besides the direct viral pathogenic role, a postinfectious inflammatory syndrome. Interestingly, at the onset of hyperinflammation, both patients showed negative SARS-CoV-2 PCR in commonly sampled sites.

## Conclusion

Despite the limited number of cases treated, our report demonstrates the occurrence of cytokine storm and hyperinflammation in some children infected by SARS-CoV-2, the late onset of this potential complication, and the effectiveness of immune-modulating therapy. This atypical presentation of pediatric patients should be always taken into consideration during the pandemic in order to ensure prompt diagnosis and appropriate therapeutic management. Additional cases and longer follow-up are needed to further validate the efficacy of our therapeutic approach.

## Data Availability Statement

The original contributions presented in the study are included in the article/supplementary material, further inquiries can be directed to the corresponding author/s.

## Ethics Statement

The studies involving human participants were reviewed and approved by IRCCS Bambino Gesù Children's Hospital. Written informed consent to participate in this study was provided by the participants' legal guardian/next of kin. Written informed consent was obtained from the minor(s)' legal guardian/next of kin for the publication of any potentially identifiable images or data included in this article.

## Author Contributions

FCC drafted the manuscript. All authors were involved in the clinical management, analyzed and interpreted the data, and discussed the results. All authors have read and approved the final manuscript.

## Conflict of Interest

The authors declare that the research was conducted in the absence of any commercial or financial relationships that could be construed as a potential conflict of interest.
